# Coming Events

**Published:** 1899-07

**Authors:** 


### COMING EVENTS.

### AMERICAN VETERINARY MEDICAL ASSOCIATION.

Place and date of next meeting: New York City, September 5, 6, and 7, 1899.

Convention Hall: Large Assembly Room of the Academy of Medicine, 17 West Forty-third Street, near Fifth Avenue.

Headquarters: Hotel Manhattan, corner Forty-second Street and Madison Avenue.

Local Committee of Arrangements: Drs. H. D. Gill (Chairman), Roscoe R. Bell, George H. Berns, E. B. Ackerman, and W. H. Pendry.

Location of Surgical Clinic: American Horse Exchange, Fiftieth Street and Broadway.

The following papers will be presented:

"Dietetics," by Dr. W. H. Dalrymple, of Louisiana.

"Acetanilid as an Antipyretic for the Horse," by Dr. R. R. Bell, of New York.

"Dairying from a Pure Milk Standpoint," by Dr. C. C. McLean, of Pennsylvania.

"Routine Manipulations and Operations," by Dr. William Herbert Lowe, of New Jersey.

"Notes on Filaria Immitis," by Dr. Benjamin McInnes, of South Carolina.

"The Tick in North Carolina," by Dr. Cooper Curtice, of North Carolina.

"The Pathology and Treatment of Azoturia," by Dr. Richard P. Lyman, of Connecticut.

"Median Neurectomy," by Dr. C. E. Clayton, of New York.

"Notes on the Healing Process in Ovariotomy," by Dr. M. H. Reynolds, of Minnesota.

"Disinfection," by Dr. E. A. A. Grange, of Michigan.

"Surgical Interference for the cure of the Cribbing Habit," by Dr. S. J. J. Harger, of Pennsylvania.

"Rheumatism in Domestic Animals," by Dr. Hermann Wellner, of New York.

"The Veterinarian of the Future," by Dr. N. S. Mayo, of Connecticut.

"Notes on Rabies," by Dr. J. M. Parker, of Massachusetts.

"Chicken Cholera," by Dr. Charles H. Higgins, of Canada.

"A Plea for a More General Use of Anæsthesia in Veterinary Surgery," by Dr. J. P. Turner, of the District of Columbia.

"Glanders in Massachusetts," by Dr. M. O'Connell, of Massachusetts.

"European Veterinary Institutions," by Dr. James B. Paige, of Massachusetts.

"Tetanus Antitoxin," by Dr. E. M. Ranck, of Pennsylvania.

"The Suppression of Tuberculosis in Pennsylvania," by Dr. Leonard Pearson, of Pennsylvania.

It now seems probable that the following will also be presented:

"Arytenoideraphy, a New Operation for the Relief of Roaring," by Dr. L. A. Merillat, of Illinois.

"The Veterinarian in Political Life," by Dr. Charles Greswell, of Colorado.

"Diseases of Horses Peculiar to the Mountain Region," by Dr. M. E. Knowles, of Montana.

"The Schmidt Treatment of Parturient Paresis," by Dr. Olof Schwartzkopf, of New York.

Kansas City will be represented at the Veterinary Conclave by Drs. R. C. Moore, John S. Buckley, and S. Stewart.

Nebraska will send a strong delegation in recognition of the Association's consideration of that State last year in holding its meeting in Omaha. Drs. J. S. Anderson, Don. C. Ayer, A. T. Peters, and H. L. Ramacciotti expect to help enjoy the entertainment promised for that Metropolitan meeting.

Dr. S. K. Spaulding, Health Commissioner of Omaha, hopes to have the pleasure of attending the meeting in New York.

Those whose untiring devotion to the cause of recognition of the veterinarian in the United States Army will be delighted to learn that a paper upon this subject will be presented at the coming meeting of the American Veterinary Medical Association by one of the pioneers in the movement, Prof. R. S. Huidekoper. Thoroughly posted as to the army veterinary service in every country in the world and our own needs and hopes, in close touch with the army service of our own country, there will be much to learn from such a paper, and many worthy suggestions for our guidance in future army legislative efforts.

---

The Silver Anniversary of the American Veterinary College now promises to be a gathering of unusual importance. The committee having in charge the work of this celebration, of so much import to the American veterinary profession, have so far matured their plans as to announce their banquet for the evening of the 5th of September, when addresses will be made by Profs. Liautard, Huidekoper, Pearson, Osgood, Gill, and many others whose acceptances are pending. President Pendry, ably assisted by Secretary Clayton, are in close touch with the more than six hundred graduates of the American Veterinary College, and there will be many old faces, strangers within the college walls for many years, who will return on this occasion to greet the leader whose earnest work, conscientious efforts, have made this institution a school among schools. A royal welcome, a warm greeting, will be given the one whom we have learned to speak of with loving devotion as "the old man."

---

The semi-annual meeting of the Illinois Veterinary Medical and Surgical Association will be held at the office of Dr. S. H. Swain, Decatur, Ill., August 3 and 4, 1899. Papers on the following subjects have been announced: "Pneumonia," "Influenza," "Ovariotomy, Canine," "Laryngitis," "Periodic Ophthalmia," "Tuberculosis, Bovine," "Dehorning, Bovine," and "Encephalitis."

---

Secretary Merillat reports the following interesting programme for the meeting of the Association of Veterinary Faculties and Examining Boards of North America, to be held in connection with the New York meeting of the American Veterinary Medical Association:

"State Examinations," by A. W. Clement.

"The Teaching of Practical Surgery," by W. L. Williams.

"Aims and Objects of an Association of Faculties," by C. Barnwell Robinson.

Subjects to be announced at the meeting, S. J. J. Harger and M. H. Reynolds.

Others to be announced later.

The doors will be open to all members and visitors of the American Veterinary Association, instead of sessions admissible to members only, as in former years.

